# The “Reverse” Latissimus Dorsi Flap for Large Lower Lumbar Defect

**DOI:** 10.1155/2012/964625

**Published:** 2012-10-03

**Authors:** Bouraoui Kotti, Olfa Jaidane, Jamel Ben Hassouna, Khaled Rahal

**Affiliations:** Department of Surgical Oncology, Salah Azaiez Institute, 36, Nelson Mandela Street, 2045 L'aouina, Tunis, Tunisia

## Abstract

The latissimus dorsi (LD) flap is one of the most common flaps used in plastic surgery based on its dominant thoracodorsal pedicle as well as free tissue transfer. The “distally based” or “reverse” fashion design has been used to repair myelomeningoceles, congenital diaphragmatic agenesis, or thoracolumbar defects. We present a case of a large lumbar defect after cancer resection covered by a combined tegument solution starring the “reverse” LD flap in its muscular version with a cutaneous gluteal flap. This flap is a safe and reliable way to cover large distal lumbar defect.

## 1. Introduction

Covering the lumbar region was always a challenge for plastic surgeons. Although different pedicled muscular and musculocutaneous flaps were described around this area, the repair of large defects is still a difficult matter [1, 2]. We present a case in which a “reverse” latissimus dorsi muscle flap was successfully used for repairing an important defect remaining after resection of a malignant recurrent tumor located in the lower lumbar region.

## 2. Case Report

**Figure 1:**
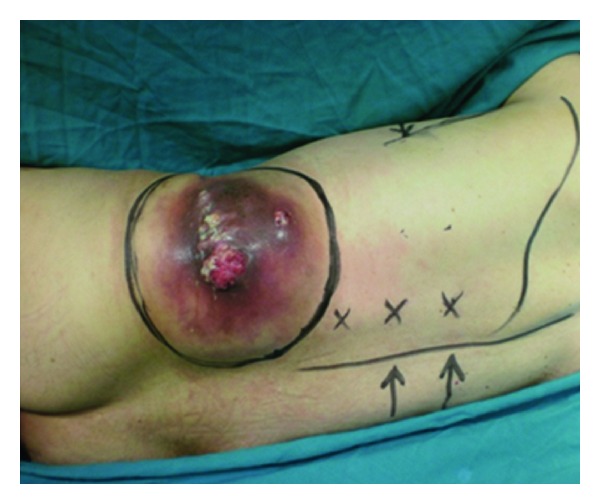
An infected recurrence on the lobotomy scar.

A 69-year-old man was referred for treatment of a massive infected tumor situated in the left lumbar region, 12 months after left nephrectomy for squamous cell carcinoma of the kidney. On clinical examination the mass was located on the lobotomy scar and measured 15 cm (Figure 1).

**Figure 2:**
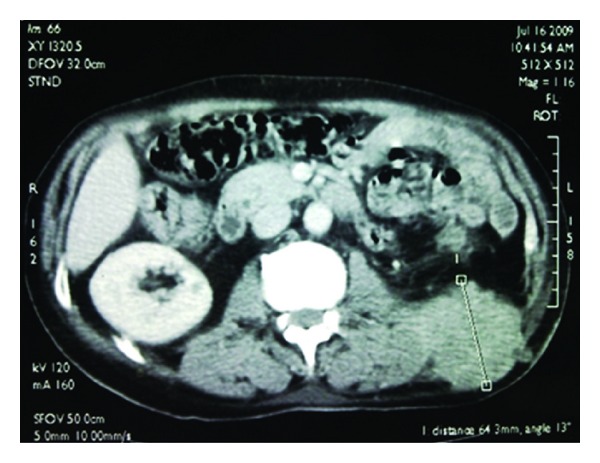
Left lumbar mass invading the iliopsoas muscle.

The abdomino-pelvic CT scan showed a mass in the left lumbar region, measuring 95 × 75 × 65 mm and invading the 11th rib. This mass extends to the ipsilateral latissimus dorsi muscle which seems to be invaded in the lower part and to the iliopsoas muscle (Figure 2).

The biopsy revealed a squamous cell carcinoma. Recurrence of the renal tumor was evoked, and surgery was indicated.

**Figure 3:**
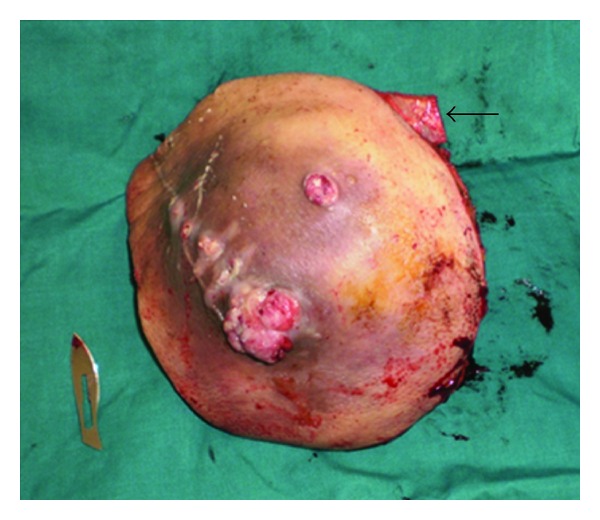
The specimen with the distal 11th rib (arrow).

**Figure 4:**
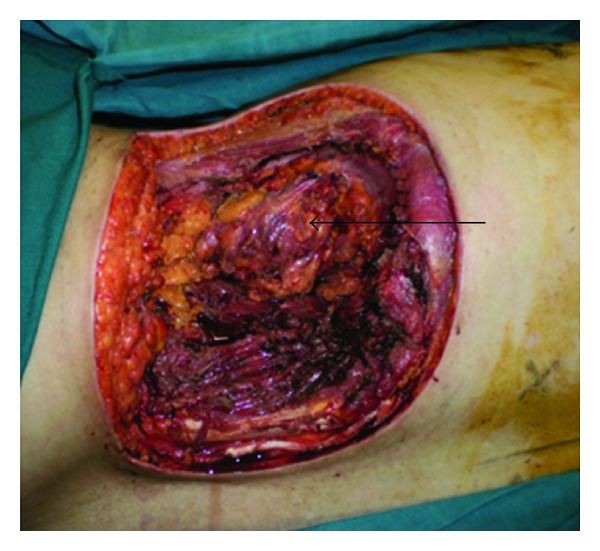
The defect showing the colonic flexure (arrow).

The tumor was removed through a circular incision, section of the lower insertion of the latissimusdorsi, the quadrates lumborum, the iliopsoas, and the two obliquus muscles. The peritoneal cavity was opened. The distal part of the 11th rib was removed with a pleural wound which was repaired. The excised tissues included also a nodule located at the superiorpart of lobotomy scar, and a second one was situated in the left retrocolic area (Figures 3 and 4).

### 2.1. The Technique

The LD outline was marked as well as its upper limit. The paraspinal perforators were outlined about 5 cm from the midline, and the penetration of the perforators through the muscle was estimated about 9 cm from the vertebral column (Figure 1). No Doppler ultra soundor arteriography was performed (not available in the center). All the benchmarks were taken based on the literature.

**Figure 5:**
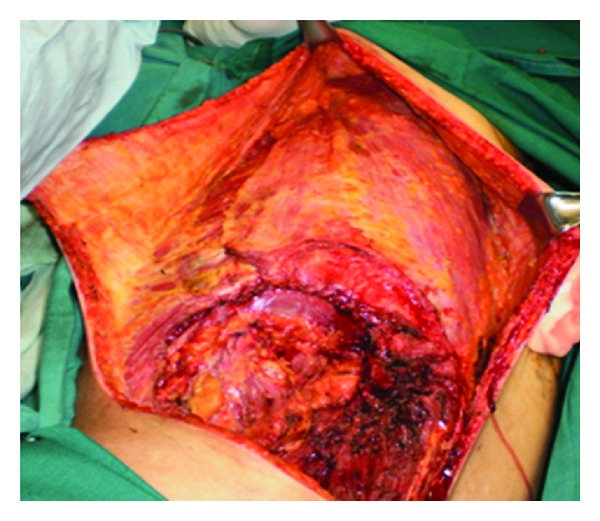
Exposure of the LD muscle.

**Figure 6:**
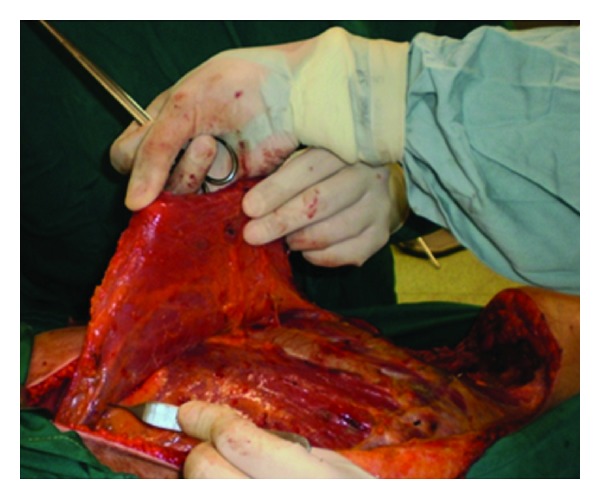
Identification of the thoracodsorsal pedicle.

An oblique incision was made from 10 cm down to the axilla to the defect. The LD was identified (Figure 5). The thoracodorsal artery, vein, and nerve were exposed, tied off and then detached (Figure 6). After section of its humerus insertion, the LD was harvested carefully in order to preserve the segmental pedicles.

**Figure 7:**
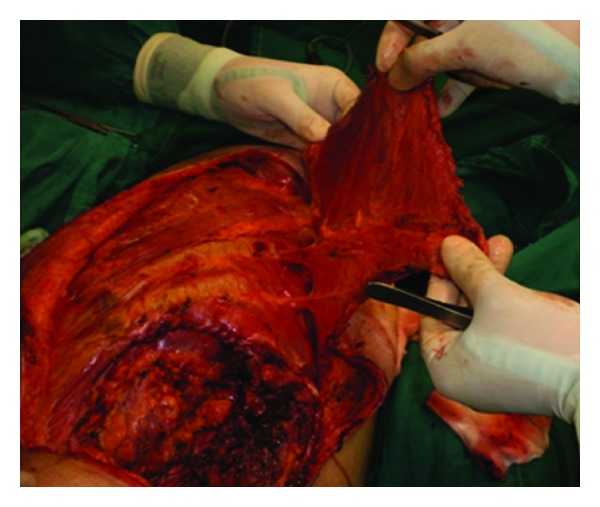
Dissection of the perforators.

We found three large perforators originating from the ninth, tenth, and eleventh intercostal pedicles, located 5 cm from the midline of the back and penetrating the muscle after 3 to 4 cm (Figure 7).

The sacrifice of the ninth pedicle was necessary to allow the LD muscle to reach the defect satisfactorily.

**Figure 8:**
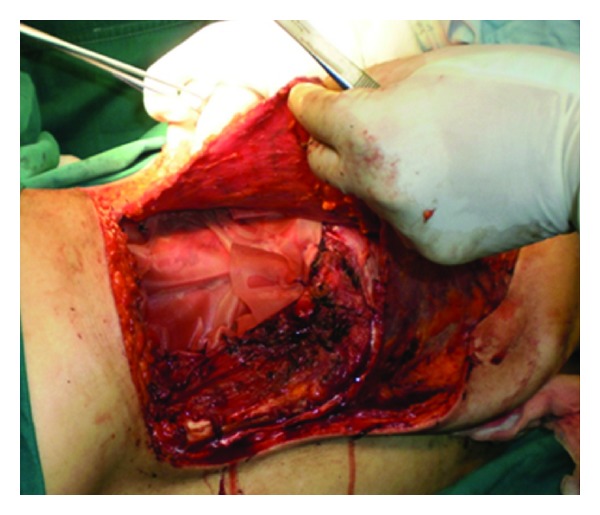
Mersilene mesh being placed.

The muscular flap was tacked with some absorbable sutures after covering the peritoneal cavity using a mersilene mesh (Figure 8). The dead space was filled up by the muscle, and a simple cutaneous rotated gluteal flap was performed to protect the sutures and strengthen the set up.

**Figure 9:**
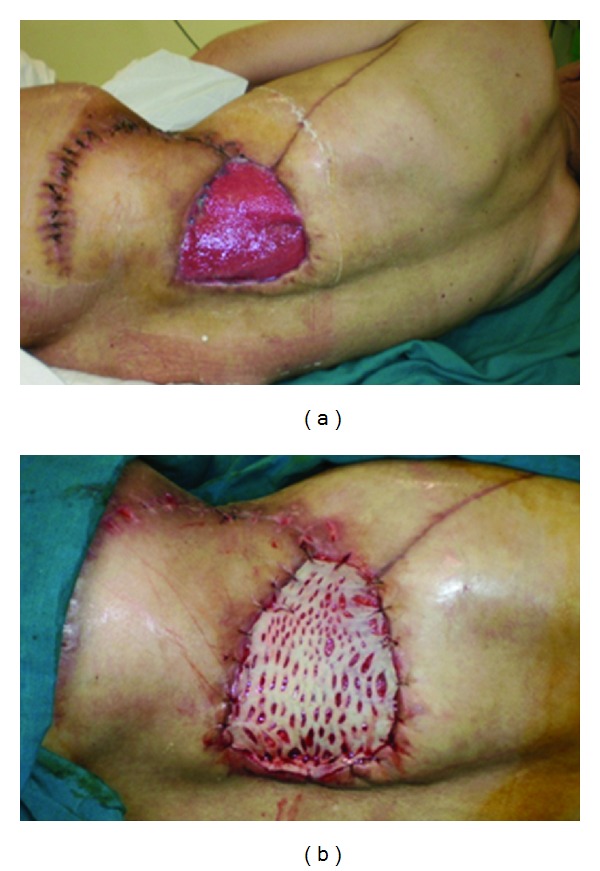
(a) Granulating wound, (b) skin graft.

Fifteen days later a good granulation tissue was obtained and a skin graft was made (Figure 9).

### 2.2. Histological Findings

On gross examination, the surgical specimen weighed 1200 g and measured 25 × 15 × 10 cm. It contained a white-mostly-necrotic nodule measuring 12 × 10 × 10 cm. On histological examination, the tumor presented a malignant squamous cell proliferation with atypia. Lateral limits of resection were not infiltrated. The posterior limit was exiguous.

The histological examination concluded to well-differentiated squamous cell carcinoma.

### 2.3. Followup

**Figure 10:**
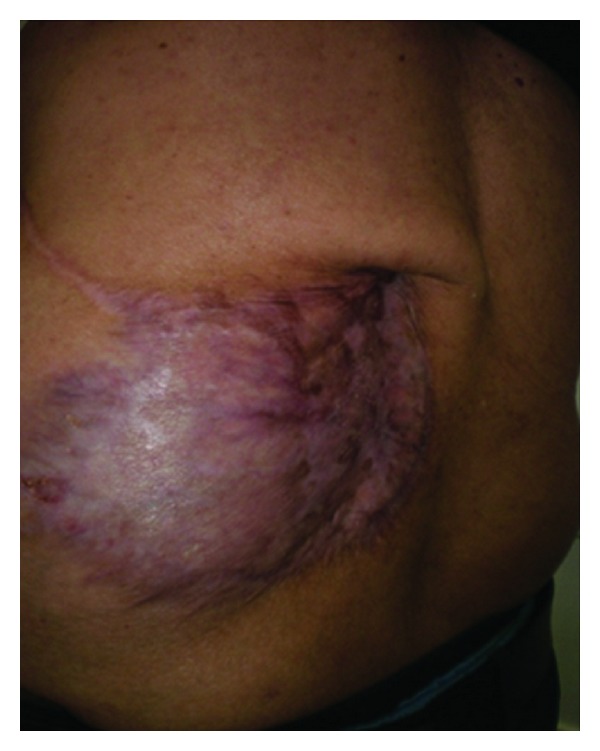
Local result after 8 months.

The postoperative course was complicated by a superficial infection treated with antibiotics and wound care and some seroma spontaneously evacuated with dressing. The coverage of this important defect was a success, and the patient was completely recovered from his wound after 6 weeks. The multidisciplinary comity took the decision to follow up the patient without any adjuvant treatment. No recurrence was observed after 8 months, but a back wall weakness was noted (Figure 10). One year later, a tumoral recurrence was diagnosed.

## 3. Discussion

Management of massive soft-tissue defects in the lumbar region is still a major challenge for plastic surgeons. This anatomical region is like a “no man's land” for us. The local solutions are rare, and the standard free tissue transfer is not an easy job, especially if the recipient vessels for microsurgical reconstruction like the gluteal arteries are far or sometimes not available.

Reverse latissimus dorsi (LD) flap has been described mainly for closure of congenital diaphragmatic agenesis, myelomeningocele and spinal cord syndrome, or some thoracolumbar defects [3–7]. But some cases for the coverage of the lower back soft-tissue loss using this flap were reported in the literature, proving by the way the possibility to reach this “no man's land” region and the reliability of the reverse LD flap to do it [2, 8, 9].

We will not discuss the oncological aspect of the treatment, but we will focus on our method to cover this massive lumbar defect. The LD has a double vascularization as described by Mathes and Nahai [10] and if it remains one of the most used flaps in plastic surgery, its “reverse” version is not so common. Described in the early eighties [3], this flap was used basically for central posterior trunk defects. Increasingly, its use was described for lower lumbar and gluteal regions [11]. Detailed anatomical studies were reported by different authors, and sometimes the results diverge even if some similarities were found. In fact McCraw et al. [12] reported that segmental perforators usually arose at the levels of the seventh, ninth, and eleventh thoracic vertebrae, approximately 8 cm from the midline.

Whereas Stevenson et al. [13] observed the presence of three large vascular pedicles originating from the ninth, tenth, and eleventh intercostal vessels, 5 cm from the midline. Grinfeder et al. [14] observed the same result for 50% of their flap dissections. The locations in our case were almost the same as described by Stevenson and Grinfeder. Although we found our perforators 5 cm from the spine, their penetration through the muscle was detected 3 to 4 cm after. This length in this cleavage plan allows some translation to the lower part, but the pivot point can be considerably increased after the sacrifice of one perforator pedicle. This sacrifice was described in different cases [2, 12, 14] and allows a rotation vector facilitating the migration for more than 5 cm in our case without altering the blood supply for the lower part of the muscle which is the most important one. The upper limit of our flap was situated 10 cm from the axilla in order to avoid distal suffering.

The exact vascular territory of each segmental pedicle is unknown [2, 14], and the skin paddle required for this big defect (25/15 cm) is too large for the reverse LD flap that we cannot avoid tampering the donor site or risking a skin necrosis. Opting for a muscle reverse LD flap with a gluteal skin flap was for us the simplest solution that can fill the dead space and cover the defect. The muscle was bleeding well even after the sacrifice of the ninth pedicle, and the granulation tissue is also a proof of viability.

We believe that the “reverse” LD flap is a good option to cover this particular region. It is simple, safe, and reliable. It also provides a backup plan like the microsurgery in case of failure.

## 4. Conclusion

We present a case of large lumbar defect covered using the latissimus dorsi flap in its reverse fashion with a satisfactory result. This pedicled flap has a good trophicity and offers an amplified rotation vector allowing reaching lower trunk areas. It is a reliable solution to solve difficult plastic tegument problems and cover large surface defects.
